# PTGES/PGE_2_ signaling links immunosuppression and lung metastasis in *Gprc5a*-knockout mouse model

**DOI:** 10.1038/s41388-020-1207-6

**Published:** 2020-02-14

**Authors:** Tong Wang, Bo Jing, Dongliang Xu, Yueling Liao, Hongyong Song, Beibei Sun, Wenzheng Guo, Jianhua Xu, Kaimi Li, Min Hu, Shuli Liu, Jing Ling, Yanbin Kuang, Tuo Zhang, Siwei Zhang, Feng Yao, Binhua P. Zhou, Jiong Deng

**Affiliations:** 1 Key Laboratory of Cell Differentiation and Apoptosis of Chinese Minister of Education, Shanghai Jiao Tong University School of Medicine, Shanghai, China; 2 Shanghai Key Laboratory for Tumor Microenvironment and Inflammation, Shanghai Jiao Tong University School of Medicine, Shanghai, China; 3 Translational Medical Research Center, Shanghai Chest Hospital, Shanghai Jiao Tong University, Shanghai, China; 4 Department of Pathology, Kunming Medical University, Kunming, Yunnan China; 5 Department of Oral and Maxillofacial–Head and Neck Oncology, the Ninth People’s Hospital, College of Stomatology, Shanghai Jiao Tong University School of Medicine, Shanghai, China; 6 Department of Oncology, Shanghai General Hospital, Shanghai Jiao Tong University School of Medicine, Shanghai, China; 7 Department of Respiratory Medicine, The Second Affiliated Hospital, Dalian Medical University, Dalian, China; 8 Department of Thoracic Surgery, Shanghai Chest Hospital, Shanghai Jiao Tong University, Shanghai, China; 9 Department of Molecular and Cellular Biochemistry, Markey Cancer Center, University of Kentucky College of Medicine, Lexington, KY USA

**Keywords:** Cancer microenvironment, Inflammation

## Abstract

Chronic inflammation has been linked to promotion of tumorigenesis and metastasis in lung. However, due to lack of a relevant animal model for characterization, the underlying mechanism remains elusive. Lung tumor suppressor gene *Gprc5a*-knockout (ko) mice are susceptible to lung inflammation, tumorigenesis and metastasis, which resembles the pathological features in human patients. Here, we showed that PTGES/PGE_2_ signaling was highly associated with lung tumorigenesis and metastasis in *Gprc5a*-ko mice. Interestingly, Ptges-knockout in mouse lung tumor cells, although reduced their stemness and EMT-like features, still formed tumors and lung metastasis in immune-deficient nude mice, but not in immune-competent mice. This suggests that the major role of PTGES/PGE_2_ signaling in tumorigenicity and lung metastasis is through immunosuppression. Mechanistically, PTGES/PGE_2_ signaling intrinsically endows tumor cells resistant to T-cell cytotoxicity, and induces cytokines extrinsically for MDSC recruitment, which is crucial for suppression of T-cell immunity. Importantly, targeting PGE_2_ signaling in *Gprc5a*-ko mice by PTGES inhibitor suppressed MDSC recruitment, restored T cells, and significantly repressed lung metastasis. Thus, PTGES/PGE_2_ signaling links immunosuppression and metastasis in an inflammatory lung microenvironment of *Gprc5a*-ko mouse model.

## Introduction

Tumor recurrence and metastasis are the major causes of cancer death [1]. Of notion, lung tumor progression and metastasis are often accompanied by inflammatory response [2]. Recently, tissue of chronic inflammation has been linked to suppressed immunity, including suppressed T cells, tumor-associated macrophages (TAM), neutrophils, and myeloid-derived suppressor cells (MDSCs). In particular, accumulated MDSCs can protect the tumor cells from immune-surveillance by constructing pre-metastatic niches. These observations suggest that, immunosuppression via MDSCs in inflammatory microenvironment plays important role in promotion of tumor progression and metastasis.

MDSCs are heterogeneous population consisting of myeloid progenitor cells and immature myeloid cells [3]. MDSC recruitment can be induced by tumor-derived chemokines and cytokines, such as granulocyte-macrophage colony-stimulating factor (GM-CSF) [4–6], G-CSF, interleukin (IL)-6 [7], IL-1β, arginase 1 (ARG1), interferon (IFN)-γ [8–10]. In clinic, immunosuppressive phenotypes, such as upregulated G-CSF, tumor-related leukocytosis, and neutrophil-to-lymphocyte (NLR), are associated with poor outcome of non-small cell lung cancer (NSCLC) patients [11]. However, the roles and mechanisms of MDSC expansion and activation are not fully understood.

Prostaglandin E2 (PGE_2_) is a key mediator of inflammation, pain, and fever [12]. PGE_2_ is one of the most abundant prostaglandins synthesized from arachidonic acid (AA). AA is oxygenated by cyclooxygenase-1 and 2 (COX-1/2) to produce PGG_2_. PGG_2_ is subsequently reduced to PGH_2_. And PGH_2_ is then converted into several prostanoids (e.g., PGF_2α_, PGD_2_, PGI_2_, TXA_2_ and PGE_2_) by a variety of synthases. PGE_2_ synthases (PGES) convert PGH to PGE_2_, the terminal product [13, 14]. The isomerization of the endoperoxide PGH_2_ to PGE_2_ is catalyzed by three different PGE synthases, cytosolic PGE synthase (cPGES) and two membrane-bound PGE synthases, PTGES and mPGES-2. cPGES and mPGES-2 are constitutive enzymes, whereas PTGES is inducible [13]. PTGES is highly upregulated in inflammatory tissues and tumors [15]. Of notion, PGE_2_ is markedly increased in many types of human cancers, including lung, colon, bladder, breast and head and neck cancer, and is often associated with a poor prognosis [16–20]. Increased PGE_2_ has a major impact on intra-tumoral inflammatory cells, promoting the immunosuppressive microenvironment [21, 22]. However, due to lack of an animal model that resembles the pathological features of human disease, the biological roles of PGE_2_ signaling in immunosuppression and lung metastasis remain unclear.

G protein coupled receptor family C group 5 type A (GPRC5A) is predominately expressed in lung tissues [23–25]. *Gprc5a*-knockout (ko) mice developed spontaneous lung adenocarcinoma [26, 27], indicating that Gprc5a is a lung tumor suppressor gene. Importantly, tumorigenesis in *Gprc5a*-ko mouse lung is associated with inflammation along with persistent activation of NF-κB, EGFR, and STAT3 signaling [26–28], which resembles the pathological features in human lung cancer. Moreover, GPRC5A is repressed in most of NSCLC and all of chronic obstructive pulmonary disease (COPD) [29]. Thus, *Gprc5a*-ko mice provide a unique animal model to study the mechanistic link between inflammatory response and tumorigenesis/metastasis in lung.

In this study, PTGES/PGE_2_ signaling was found greatly enhanced in lung tumorigenesis and metastasis in *Gprc5a*-ko mouse model. We found that, the major mechanism in promotion of lung metastasis is through immunosuppression by PTGES/PGE_2_ signaling.

## Results

### PTGES/PGE_2_ signaling is activated in lung tumor cells of *Gprc5a*-ko mouse model

**Fig. 1:**
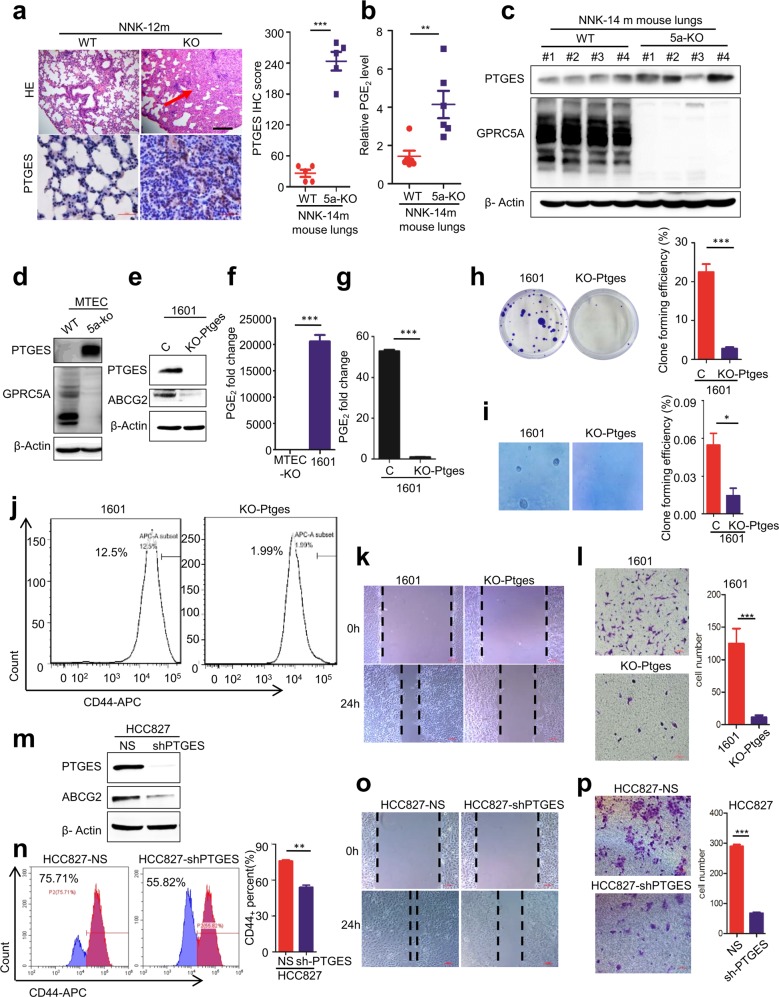
PTGES/PGE_2_ signaling is activated in lung tumor cells of *Gprc5a*-ko mouse model. **a** Representative images of lung tissues stained with H&E and IHC for Ptges of *Gprc5a*-ko-NNK-12m mice lung tissue, scale bar = 400 μm (mean ± SD (*n* = 5)). **b** Metabonomics analysis of the secretion of PGE_2_ from NNK-14m-WT and NNK-14m-KO mouse lung tissues (*n* = 6). **c** Western blotting analysis of PTGES protein levels in NNK-14m-WT and NNK-14m-KO mouse lung tissues. **d** Immunoblot analysis of cell lysates from *Gprc5a*-ko or *Gprc5a*-wt MTEC with PTGES and GPRC5A antibodies as indicated. **e** Western blotting analysis of PTGES and ABCG2 protein levels in SJT-1601 cells with and without knockout of Ptges via CRISPR/Cas9 technology. **f** ELISA kit analysis of the secretion of PGE_2_ in MTEC-KO and SJT-1601 cells (*n* = 3). **g** ELISA kit analysis of the secretion of PGE_2_ from SJT-1601 and SJT-1601-ko-Ptges cell lines (*n* = 3). **h** Colony formation assay of SJT-1601 control and SJT-1601-ko-Ptges cells from 3 separate experiments. **i** Measurement of tumorsphere formation from 3 separate experiments in SJT-1601 and SJT-1601-ko-Ptges cells. Graph shows the mean ± SD percentage; scale bar = 100 μm. **j** Flow cytometry analysis of CD44^+^ in SJT-1601 and SJT-1601-ko-Ptges cells. **k** Wound healing and migration analysis of SJT-1601 cells with or without PTGES silencing at 24 hours. Scale bar = 100 μm. **l** Migration of SJT-1601 cells with or without PTGES silencing analyzed by Transwell migration assay (mean ± SD (*n* = 3)), scale bar = 100 μm. **m** Western blotting analysis of PTGES and ABCG2 protein levels in HCC827 and HCC827-shPTGES cell lines. **n** Flow cytometry analysis of CD44^+^ in HCC827 and HCC827-shPTGES cells (mean ± SD (*n* = 3)). **o** Wound healing and migration analysis of HCC827 cells with or without PTGES silencing at 24 hours. Scale bar = 100 μm. **p** Migration of HCC827 cells with or without PTGES silencing analyzed by Transwell migration assay (mean ± SD (*n* = 3)), scale bar = 100 μm. *p < 0.05; **p < 0.01; ***p < 0.001.

Previously, *Gprc5a*-ko mice were shown to develop spontaneous lung cancer in 1.5 to 2 years [27], and lung tumorigenesis was associated with pulmonary inflammation [30]. Treatment with tobacco carcinogen NNK-induced lung tumor development in 100% *Gprc5a*-ko (KO) mice (10/10) but 0% wild-type mice (0/10) in 12 months (Fig. 1a) [27]. To determine which pathways are important in this lung tumor model, we performed RNAseq and pathway analysis on normal mouse tracheal epithelial cells (MTEC) derived from wild-type (WT) mouse and *Gprc5a*-ko (KO) one. The results showed that arachidonic acid metabolism was one among the significantly upregulated pathways. Importantly, comprehensive analysis of the metabolites showed that PGE_2_ is greatly upregulated in tumor-bearing NNK-14m-KO mouse lungs, compared to the lungs of NNK-14m-WT mice (Fig. 1b, Supplementary Fig. S1). Consistently, immunoblot showed that PTGES was significantly upregulated in NNK-14m-KO mouse lungs, compared to those of NNK-14m-WT ones (Fig. 1c). Consistently, IHC staining showed that PTGES was upregulated in lung tumor tissues in NNK-14m-KO mouse lungs compared to lung tissues of NNK-14m-WT ones (Fig. 1a). Taken together, these results suggest that upregulated PTGES/PGE_2_ signaling is correlated with lung tumorigenesis in *Gprc5a*-ko mouse model.

Next, we asked if there is a causal relationship between GPRC5A-deficiency and PTGES upregulation. Immunoblot analysis showed that Ptges was greatly upregulated in KO-MTEC compared to that in WT-MTEC (Fig. 1d). Of notion, SJT-1601 cells, a lung tumor cell line derived from a *Gprc5a*-ko mouse lung tumor, were also expressing high level of Ptges (Fig. 1e), suggesting that Ptges overexpression contributes to tumorigenesis in *Gprc5a*-ko mouse lungs. To determine the biological impact of PTGES overexpression in lung cancer cells, we established 1601-ko-Ptges cells by knockout Ptges in SJT-1601 cells via the CRISPR/Cas9 system (Fig. 1e). Ptges-ko in 1601 cells abolished the expression of Ptges, and the production of PGE_2_ (Fig. 1f, g), reduced colony formation in colonogenic assay (Fig. 1h, Fig. S2) and anchorage-independent growth (Fig. 1i). Consistently, CD44^+^ cells, a stem cell-enriched subpopulation, in 1601-shPtges cells (1.99%) were dramatically reduced compared to that in parental 1601 cells (12.5%) (Fig. 1j). Functionally, SJT-1601-ko-Ptges cells exhibited reduced migration (Fig. 1k) and invasion (Fig. 1l) compared to that of parental SJT-1601 cells. To extend the analysis further, we established HCC827-shPTGES cell lines via shRNA, in human NSCLC cell line HCC827 cells that express relatively high levels of PTGES (Fig. 1m). PTGES knockdown also reduced CD44^+^ subpopulation in HCC827-sh-PTGES cells (55.8%) compared to parental HCC827 cells (75.7%) (Fig. 1n). Consistently, migration and invasion of HCC827-sh-PTGES cells was significantly reduced compared to those of HCC827 cells (Fig. 1o, p). Taken together, these results suggest that the PTGES/PGE_2_ pathway is essential for the enhanced stemness and pro-metastatic features in human NSCLC and mouse lung cancer cells.

### PTGES/PGE_2_ signaling links immunosuppression and tumorigenicity/lung metastasis in immune-competent mice

**Fig. 2:**
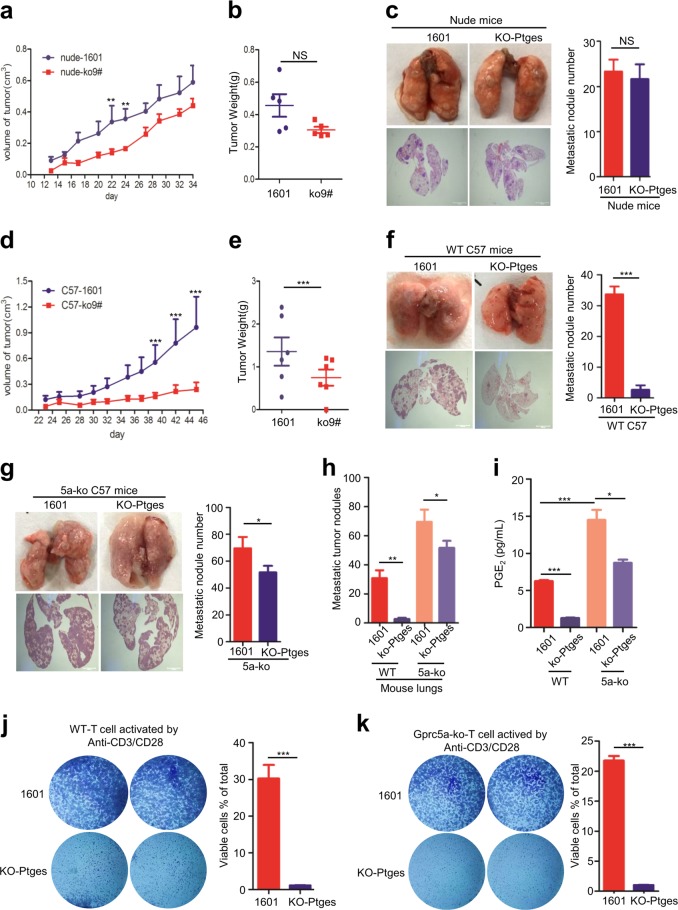
PTGES/PGE_2_ signaling links immunosuppression and tumorigenicity / metastasis in lungs of the immune-competent mice. **a** Mouse lung tumor SJT-1601 and SJT-1601-ko-Ptges cells were s.c. injected into nude mice, and tumor size was measured as indicated. **b** Tumors weight was measured as indicated (mean ± SD (*n* = 5)). **c** Nude mice were infected through the tail vein with 5 × 10^**4**^ SJT-1601 or SJT-1601-ko-Ptges cells. Lungs were removed from the mice at 3 weeks after inoculation. The experimental scheme is presented. Representative images of lung tissues stained with H&E are presented (bottom). Scale bar = 1000 μm. Number of metastatic lesions in the lungs (*n* = 5). **d** Mouse lung tumor SJT-1601 and SJT-1601-ko-Ptges cells were s.c. injected into C57BL/6 mice, and tumor size was measured as indicated. **e** Tumors weight was measured as indicated. **f** C57BL/6 mice were infected through the tail vein with 5 × 10^**5**^ SJT-1601 or SJT-1601-ko-Ptges cells. Lungs were removed from the mice at 3 weeks after inoculation. The experimental scheme is presented. Representative images of lung tissues stained with H&E are presented (bottom). Scale bar = 1000 μm. Number of metastatic lesions in the lungs (*n* = 6). **g** *Gprc5a*-ko mice were infected through the tail vein with 5 × 10^**5**^ SJT-1601 or SJT-1601-ko-Ptges cells. Lungs were removed from the mice at 3 weeks after inoculation. The experimental scheme is presented. Representative images of lung tissues stained with H&E are presented (bottom). Scale bar = 1000μm. Number of metastatic lesions in the lungs (*n* = 5). **h** Numbers of metastatic nodules in f and g. **i** ELISA kit analysis of the secretion of PGE_2_ in mice lung tissues (*n* = 3). **j** C57-WT T-cell-mediated tumor cell killing assay in SJT-1601and SJT-1601-ko-Ptges cells. **k** *Gprc5a*-ko T-cell-mediated tumor cell killing assay in SJT-1601and SJT-1601-ko-Ptges cells (*n* = 3). ns, not significant; *p < 0.05; **p < 0.01; ***p < 0.001.

To determine the biological impact of PTGES/PGE_2_ signaling on tumor cells in vivo, we examined the tumorigenicity and lung metastasis of these cells in both immune-deficient nude mice and immune-competent C57BL/6 mice. SJT-1601 vs SJT-1601-ko-Ptges cells were s.c. injected in both nude mice and C57BL/6 mice, and the tumor sizes in these groups were measured. Interestingly, there was no significant difference between the tumorigenicity of SJT-1601 cells and that of SJT-1601-ko-Ptges cells in nude mice (Fig. 2a, b). Similarly, lung metastasis via tail vein injection resulted in similar metastatic nodules (Fig. 2c). However, the tumorigenicity and lung metastasis of 1601-ko-Ptges cells was dramatically suppressed compared to that of SJT-1601 cells in immune-competent C57BL/6 mice (Fig. 2d-f). These results suggest that the major impact of PTGES/PGE_2_ signaling of tumor cells on tumorigenicity and lung metastasis is through immunosuppression.

*Gprc5a*-deficienct mice were prone to lung tumorigenesis, which was associated with chronic inflammation in lungs [27]. To determine the effect of inflammatory microenvironment on lungs metastasis, we then examined the metastasis of 1601 and 1601-ko-Ptges in *Gprc5a*-ko mice. 1601-ko-Ptges cells formed more metastatic nodules in *Gprc5a*-ko mouse lungs compared to those in wild-type mice although still less than 1601 cells (Fig. 2g, h). These results suggest that the immune surveillance in *Gprc5a*-ko mouse lungs is greatly compromised. Noticeably, the pattern of the lung metastatic nodules of these groups (Fig. 2h) were strongly correlated with the pattern of the PGE_2_ levels in these groups (Fig. 2i), suggesting that PGE_2_ signaling contributes to the susceptibility to lung metastasis, or the immunosuppression. To determine the role of PTGES/PGE_2_ signaling of tumor cells on T-cell immunity, we performed cytolytical T-cell assay on 1601 and 1601-ko-Ptges cells. Activated T lymphocytes did not kill parental 1601 cells, but killed 1601-ko-Ptges cells (Fig. 2j, k). This suggests that, PTGES/PGE_2_ signaling endows tumor cells resistant to T-cell-mediated cytotoxicity.

### Tumor cell-derived PGE_2_ induces polarization of type II macrophages

**Fig. 3:**
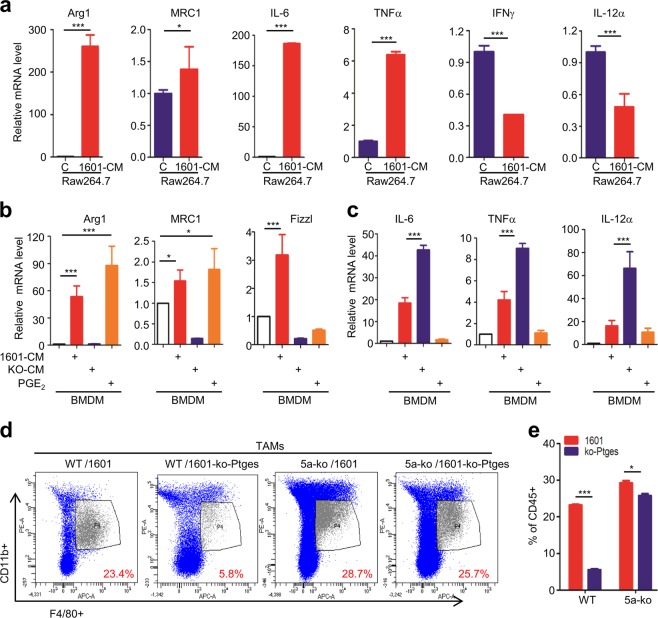
Tumor cell-derived PGE_2_ induces polarization of type II macrophages. **a** The mRNA level of relative expression of Arg1, Mrc1, TNFα, IL-6, IFNγ and IL12α in RAW264.7 cells with or without SJT-1601 condition medium. **b**, **c** The mRNA level of genes that are related to TAMs in BMDMs with the condition medium of SJT-1601 and SJT-1601-ko-Ptges cells and PGE_2_. **d** Representative FACS images for stained TAM in tumor lung tissues (*n* = 5). **e** Statistical analysis of d. ns, not significant; *p < 0.05; **p < 0.01; ***p < 0.001.

Macrophages have 2 types: M1 type promotes anti-tumor immunity, whereas M2 type promotes tumor progression. Tumor-associated macrophages (TAMs) are M2 macrophages, playing an important role in tumor progression and metastasis [31]. The phenotype of M1 and M2 type can be differentiated by the biomarker expressing, such as IFNγ, IL12α in M1, and Arg1, MRC1, IL-6, in M2 type. To determine the extrinsic roles of PTGES/PGE_2_ signaling in regulation of the immunity, we next examined the effects of the PGE_2_-containing condition media on differentiation of macrophages. Co-culture of macrophage cells, RAW264.7, with the conditioned medium (CM) from SJT-1601 cells (1601-CM), induced M2 markers, Arg1, MRC1, and enhanced the expression of pro-inflammatory cytokines, TNFα, IL-6; whereas significantly reduced the expression of M1 markers, IFNγ and IL-12α (Fig. 3a). These results suggest that certain released factors in the CM of tumor cells 1601 induce polarization of M2 phenotype. Similarly, co-culture of bone marrow-derived macrophages (BMDM) with 1601-CM greatly increased M2 markers, Arg1, MRC1 and Fizzl (Fig. 3b). However, co-culture of BMDM with the CM from 1601-ko-Ptges cells (KO-CM) lost all of these activities (Fig. 3b), suggesting that Ptges product PGE_2_ is responsible for the effects. Indeed, addition of exogenous PGE_2_ largely restored the expression of M2 markers, Arg1 and MRC1 although not Fizzl (Fig. 3b). These results strongly support that, PGE_2_ is mainly, although not totally, responsible for the effects. IL-6 and TNFα are pro-inflammatory cytokines, but they are also used as M1 markers in some conditions [32]. Interestingly, co-culture of BMDM with the CM from 1601-ko-Ptges cells induced higher levels of IL-6, TNFα and IL-12α than co-culture with CM from 1601 cells; whereas addition of exogenous PGE_2_ did not induce these gene products (Fig. 3c). These suggest that, in addition to PGE_2_, there are other released factors in the CM from 1601 cells that are responsible for the induction of IL-6, TNFα, IL-12α, or other immunosuppressive effects.

The ratio of macrophages in lavage fluid is generally correlated with the amount of macrophages in lung tissue, which is in proportion to the tumor burden in lung tissues. FACS analysis of TAMs in lavage fluids from mice of these groups showed that C57-WT mice injected with SJT-1601, contained more M2 macrophages, the subpopulation with marker F4/80 and CD11b + positive, than those injected with SJT-1601-ko-Ptges cells; moreover, *Gprc5a*-ko mice injected with either 1601 or 1601-ko-Ptges contained high ratio of M2 macrophages (Fig. 3d). Similar results were observed in myeloid cell population (CD45^+^) (Fig. 3e). The patterns of TAMs and CD45 + cells are very similar to that of lung metastatic nodules in these groups (Fig. 2h). Taken together, these results suggest that, PGE_2_ from tumor cells can induce polarization of M2 macrophages in vitro; and PTGES/PGE_2_ signaling-upregulated TAMs and myeloid cells are strongly correlated with immunosuppression and lung metastasis in *Gprc5a*-ko mouse lungs.

### PTGES/PGE_2_-mediated recruitment of MDSCs is essential for immune suppression

**Fig. 4:**
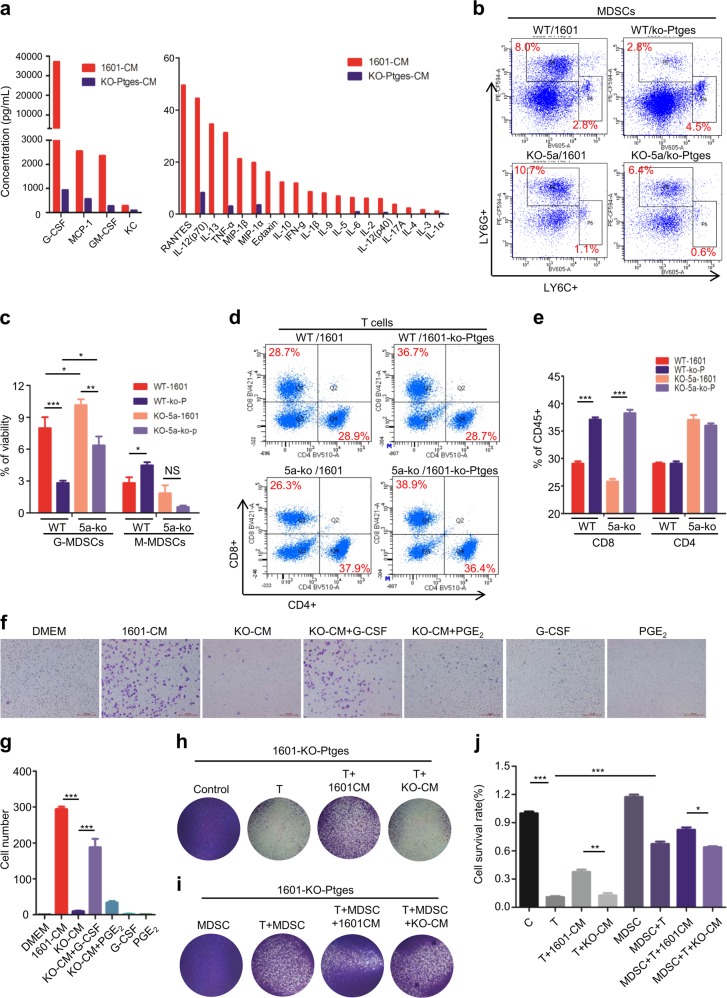
PTGES/PGE_2_-mediated recruitment of MDSCs is essential for immune suppression. **a** Twenty three-factor detection of supernatant medium from SJT-1601 and SJT-1601-ko-Ptges cells. **b** Representative FACS images for G-MDSCs and M-MDSCs staining in C57BL/6-WT and *Gprc5a*-ko mice tumor lung tissues (*n* = 5). **c** Statistical analysis of **b**. **d** Representative FACS images for T cells staining in C57BL/6-WT and *Gprc5a*-ko mice tumor lung tissues (*n* = 5). **e** Statistical analysis of **d**. **f**, **g** Recruit MDSCs experiment under different culture conditions by transwell experiments (*n* = 3). **h**–**j** T-cell-mediated cytotoxicity was assessed in SJT-1601-ko-Ptges cells with or without the condition medium of SJT-1601 and SJT-1601-ko-Ptges cells (*n* = 3). ns, not significant; *p < 0.05; **p < 0.01; ***p < 0.001.

To determine which released factors from Ptges-upregulated tumor cells are mainly mediating immunosuppression, we examined a panel of cytokines and chemokines in the CMs of 1601 cells vs those of 1601-ko-Ptges cells. The assay via Bio-plex MAGPIX system showed that most cytokines and chemokines in the CM of parental 1601 cells were significantly higher than those in the CM of Ptges-ko 1601 cells (Fig. 4a). Of notion, G-CSF, MCP-1, GM-CSF and TNFα were dramatically increased at high level in the CM of SJT-1601 cell cultures compared to that of SJT-1601-ko-Ptges cell cultures (Fig. 4a, left). This suggests that PTGES/PGE_2_ signaling is essential for production of these cytokines and chemokines.

As G-CSF, GM-CSF, MCP-1, and TNFα are known to be important cytokines for recruitment of MDSCs [33–35], we then examined the recruitment of MDSCs in vivo. FACS analysis showed that, more G-MDSCs were recruited in mice injected with 1601 cells than those injected with 1601-ko-Ptges cells (8.0% vs 2.8% in WT mice; and 10.7% vs 6.4% in *Gprc5a*-ko mice), and more G-MDSCs were recruited in *Gprc5a*-ko mice than in C57-WT ones (10.7% vs 8.0% for 1601 cells; and 6.4% vs 2.8% for 1601-ko-Ptges cells) (Fig. 4b, c). These results suggest that, (i) active PTGES/PGE_2_ signaling in tumor cells contributes the increased recruitment of MDSCs; and (ii) G-MDSCs recruitment is enhanced in inflammatory microenvironment of *Gprc5a*-ko mouse lungs compared to C57-WT mouse lungs. In other words, inflammatory microenvironment of *Gprc5a*-ko mouse lungs is immunity-compromised, or immunosuppressive, compared to that of wild-type ones.

T cells are the major components of adaptive immunity against tumor cells. Reduced T-cell infiltration is correlated with immunosuppression, tumor progression and metastasis [36]. To determine the status of T-cell infiltration in vivo, we examined the subpopulations of CD8 and CD4 T cells from lungs by FACS assay. The results showed that CD8^+^ T cells were reduced in C57-WT and *Gprc5a*-ko mouse lungs that received 1601 cells, compared to those received 1601-ko-Ptges cells (28.7% vs 36.7% in WT mice; and 26.3% vs 38.9% in *Gprc5a*-ko mice) (Fig. 4d, e). This suggests that PTGES/PGE_2_ signaling in the metastatic tumor cells is critical for inhibition of CD8^+^ T-cell infiltration in lungs. Since the effect of PGE_2_ signaling on CD4^+^ T-cell infiltration was minimal (Fig. 4e), PGE_2_ signaling is mainly acting on CD8^+^ T-cell infiltration.

MDSCs are known for its inhibitory effect on T-cell cytotoxicity or CD8 T-cell activity. Thus, the released factors that are essential for recruitment of MDSCs will be critical. Because G-CSF was the most highly expressed and upregulated cytokine among all cytokines in 1601-CM compared to 1601-ko-Ptges-CM (Fig. 4a, left), we then examined the role of G-CSF on MDSC recruitment by in vitro infiltration assay. The results showed that 1601-CM induced MDSC recruitment whereas KO-CM did not, suggesting the products of the PTGES/PGE_2_ pathway are required for the effects. Importantly, addition of exogenous G-CSF in presence of KO-CM largely restored MDSC-recruitment, whereas addition of exogenous PGE_2_ did not (Fig. 4f, g). Interestingly, addition of exogenous G-CSF or PGE_2_ alone did not increase MDSC-recruitment (Fig. 4f, g). In addition, Gprc5a deletion did not significantly affect the functions of MDSC and CD8 cells (Supplementary Figs. S3, S4). These suggest that G-CSF, in combination with other released factors, exerts the full activity on MDSC-recruitment. Thus, PGE_2_ signaling-mediated MDSC recruitment is through induction of G-CSF and other released factors.

To determine the effects of MDSCs on T-cell activity, we performed in vitro CTL assay. T cells killed more 1601-ko-Ptges cells than 1601 cells, whereas addition of exogenous MDSCs greatly inhibited T-cell-mediated cytotoxicity in 1601-ko-Ptges tumor cells (Fig. 2j, Fig. 4h, i). Interestingly, addition of 1601-CM, but not KO-CM, inhibited T-cell activity, whereas addition of 1601-CM plus MDSCs further enhanced the inhibition (Fig. 4i, j). These suggest that certain released factors from 1601 cells by the PTGES/PGE_2_ pathway contribute to MDSC-mediated inhibition of CD8^+^ T-cell activity. Because the metastasis of 1601 and 1601-ko-Ptges cells were similar in nude mice that have macrophages and NK cells (Fig. 2c), the effect of macrophage and NK on inhibition of lung metastasis is presumably to be negligible or via adaptive immunity. Taken together, PTGES/PGE_2_ signaling plays two critical roles in lung metastasis: (i) the intrinsic role: PTGES/PGE_2_ signaling endows tumor cells resistant to T-cell cytotoxicity; and (ii) the extrinsic roles: PGE_2_ directly induces polarization of M2 macrophages, and through induction of cytokines/chemokines, such as G-CSF, PTGES/PGE_2_ signaling enhances MDSC recruitment, which inhibits T-cell cytotoxicity. Thus, PTGES/PGE_2_ signaling is a promising target for inhibiting lung metastasis.

### PTGES targeting restores host immunity and inhibits lung metastasis

**Fig. 5:**
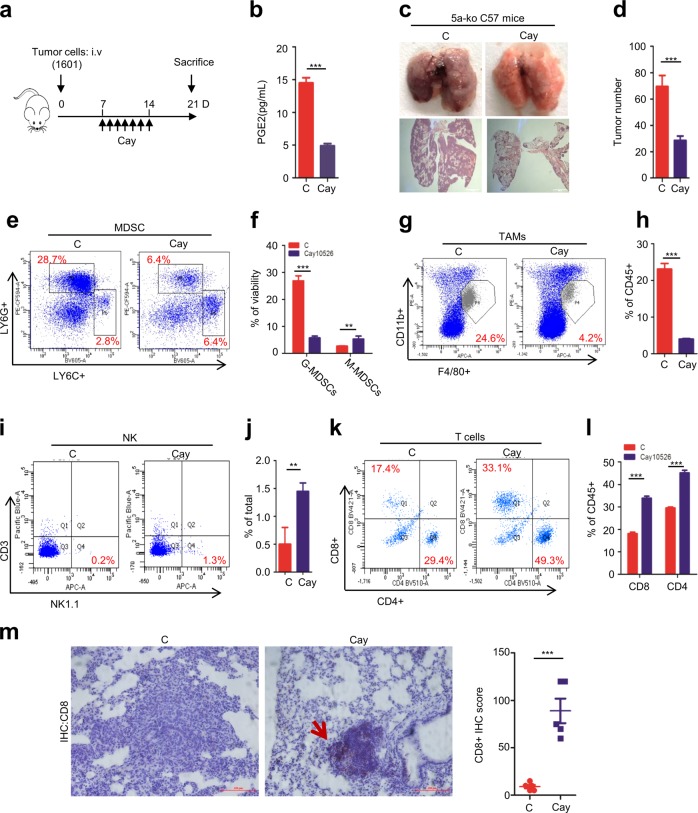
PTGES targeting restores host immunity, and inhibits lung metastasis. SJT-1601 cells were i.v. injected into *Gprc5a*-ko mice and Cay10526 was i.p injected according to the prescribed method (*n* = 5). **a** Model diagram of targeting Ptges with Cay10526 in *Gprc5a*-ko mice lung metastasis model. **b** ELISA kit analysis of the secretion of PGE_2_ in mice lung tissues. **c** Representative images of lung tissues stained with H&E. **d** Number of metastatic nodules of b. **e** Representative FACS images for G-MDSCs and M-MDSCs staining in tumor lung tissues. **f** Statistical analysis of **e**. **g** Representative FACS images for stained TAMs cells in tumor lung tissues. **h** Statistical analysis of **g**. **i** Representative FACS images for stained NK cells in tumor lung tissues. **j** Statistical analysis of i. **k** Representative FACS images for stained T cells in tumor lung tissues. **l** Statistical analysis of **k**. **m** Representative images of lung tissues with IHC staining for CD8 in control and Cay10526 treatment mice lung tissue, scale bar = 400 μm. ns, not significant; *p < 0.05; **p < 0.01; ***p < 0.001.

Due to the critical role of PTGES/PGE_2_ signaling in immunosuppression, we asked whether targeting PTGES/PGE_2_ signaling could inhibit lung metastasis efficiently in vivo. *Gprc5a*-ko mice were i.v. injected with lung tumor cells 1601, seven days later, mice of treatment group were received Cay10526, an inhibitor of PTGES, via i.p. daily injection at 5 mg/kg for 7 days. Mice of all groups were sacrificed at day 21 (Fig. 5a). PGE_2_ was significantly repressed following the treatment (Fig. 5b). The therapeutic dose of Cay10526 used in this study is 10 times less than that used (50 mg/kg) in literature, suggesting the inhibitory effect on metastasis by PTGES inhibitor is indirect [37]. In fact, no cytotoxic effect was observed in 1601 cells treated with Cay10526 up to 50 μM for 3 days (Supplementary Fig. S5). Lung tumor metastasis was significantly suppressed in treatment group compared to untreated control group (Fig. 5c, d). These suggest that PTGES/PGE_2_ signaling is critical for lung metastasis in *Gprc5a*-ko mice. Mechanistically, MDSCs, F4/80^+^/CD11b^+^ macrophages, or TAMs, were all significantly suppressed following treatment with Cay10526 (Fig. 5e-h). In contrast, NK, and CD8^+^, CD4^+^ T cells were significantly restored following Cay10526 treatment (Fig. 5i-l). Also, CD8^+^ expression were higher in tumor tissues following Cay10526 treatment (Fig. 5m). Taken together, these results suggest that PTGES targeting, via PTGES inhibitor Cay10526, suppresses MDSC recruitment, and restores T-cell immunity, which contributes to repression of lung metastasis in *Gprc5a*-ko mouse model.

### Dysregulated PTGES is inversely correlated with GPRC5A and CD8^+^ T lymphocyte infiltration in NSCLCs

**Fig. 6:**
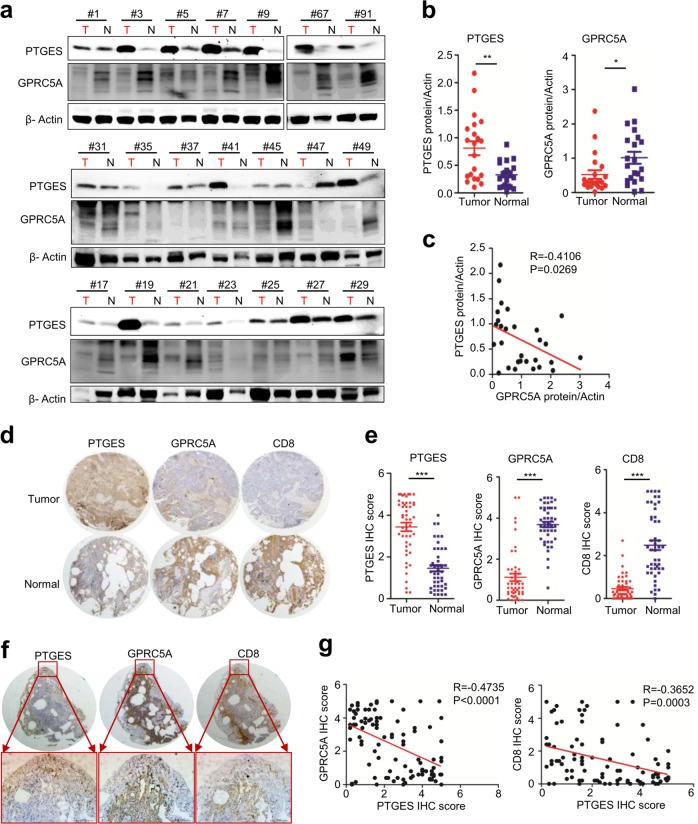
Dysregulated PTGES is inversely correlated with GPRC5A and CD8^+^ T lymphocyte infiltration in NSCLCs. **a** A subset of lung tumor and normal tissues were subjected to western blotting to examine the PTGES and GPRC5A protein levels (*n* = 21 pairs). **b** Western blotting scores of PTGES and GPRC5A from the tissues as indicated. **c** PTGES and GPRC5A expression patterns being highly negative correlated in the samples. **d** Representative staining of GPRC5A, PTGES and CD8 in lung carcinoma and peritumoral lung tissues of patients from tissue chip samples (*n* = 50 pairs). **e** IHC scores of GPRC5A, PTGES and CD8 from the tissue chip as indicated. **f** Representative images of human lung tissues chip including tumor and adjacent normal tissues stained with IHC for GPRC5A, PTGES and CD8. **g** Correlation expression analysis of PTGES, GPRC5A and CD8 in human lung tissues chip. ns, not significant; *p < 0.05; **p < 0.01; ***p < 0.001.

*Gprc5a*-ko mouse lungs are prone to inflammatory stimuli, whereas GPRC5A is repressed in most of NSCLC and all of COPD [29]. Thus, suppressed GPRC5A is a candidate biomarker of the inflammatory status of lung. To explore the relationship between GPRC5A and PTGES, we measured their expression, via immunoblot, in a panel of 21 pairs of NSCLC and adjacent normal lung tissues. The results showed that, PTGES was significantly upregulated in 15 of 21 (71.4%) lung tumor tissues, whereas GPRC5A is significantly downregulated 13 of 21 (61.9%) lung tumor tissues, compared to normal lung tissues (Fig. 6a, b). GPRC5A and PTGES expression were inversely correlated (*R* = −0.4735, *P* = 0.0269) (Fig. 6c). To extend this analysis further, we further examined, by IHC staining assay, GPRC5A and PTGES in 50 pairs of lung tissues from normal and tumor tissues. PTGES level was significantly higher in tumors than those in adjacent normal tissues, whereas GPRC5A and CD8^+^ expression were higher in adjacent normal than tumor tissues (Fig. 6d, e). Of notion, even in the same lung tumor tissues, those areas with high PTGES were low in CD8 and GPRC5A; whereas those areas with low PTGES were high in CD8 and GPRC5A (Fig. 6f). Statistical analysis indicated that PTGES was inversely correlated with GPRC5A in NSCLC (*R* = −0.4735, *P* < 0.0001) (Fig. 6g). And, PTGES was also inversely correlated with CD8 in expression (*R* = −0.3652, *P* = 0.0003) (Fig. 6g). Collectively, these results support the notion that inflammatory lungs are associated with activated PTGES, whereas activated PTGES/PGE_2_ signaling inhibits the infiltration of CD8^+^ T cells in NSCLCs.

### Upregulated PTGES in lung cancer patients predicts poor overall survival

**Fig. 7:**
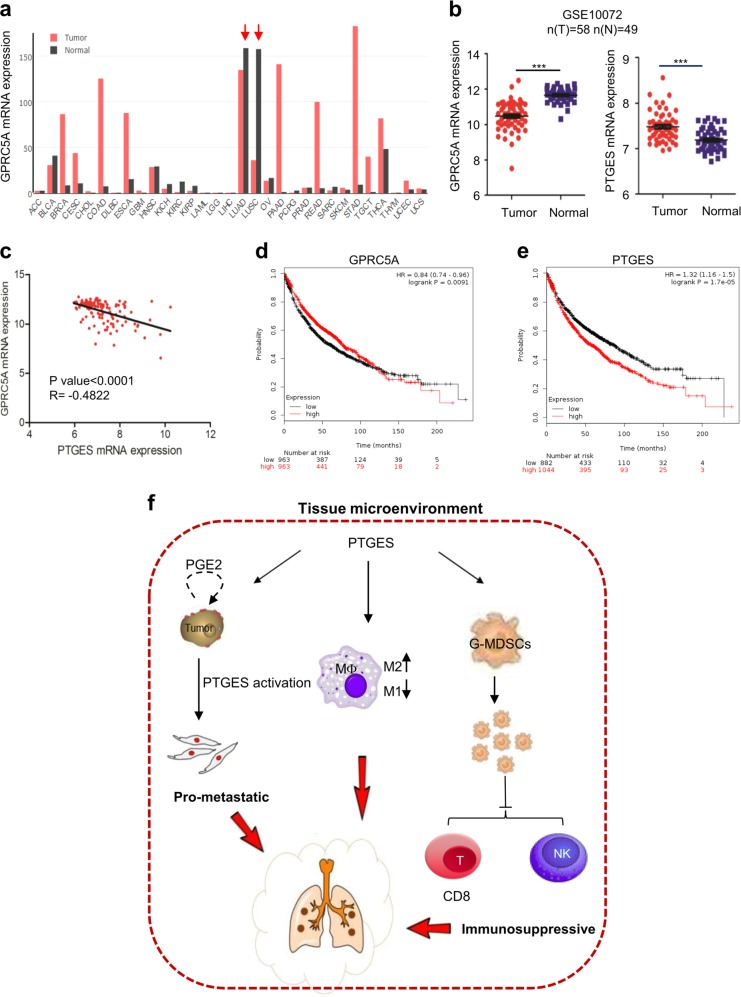
GPRC5A lowexpression is associated with poor prognosis in non-small cell lung cancer (NSCLC). **a** Comparison of mRNA expression levels of GPRC5A in cancer and adjacent normal tissues of different cancer types in TCGA database. **b** GPRC5A and PTGES mRNA expression levels in human NSCLC tumor samples compared with those in adjacent normal tissues in the GEO database (GSE10072). **c** Correlation expression analysis of GPRC5A and PTGES in human lung tissues in the GEO database (GSE10072). **d** GPRC5A expression was negatively correlated with patient overall survival in TCGA database. **e** PTGES expression was negatively correlated with patient overall survival in TCGA database. **f** Schematic depiction of the proposed action and mechanisms of PTGES-mediated immunosuppressive properties in lung cancer. ns, not significant; *p < 0.05; **p < 0.01; ***p < 0.001.

To extend the analysis further, we also analyzed GPRC5A mRNA levels in human tumor samples in TCGA using the GEPIA database. The results showed that, GPRC5A expression (black bar) was high in lung tissues, but significantly reduced in human lung adenocarcinoma (LUAD) and lung squamous cell carcinoma (LUSC) although its expression in other type of tumors (red) was upregulated (Fig. 7a). Consistently, GEO database (GSE10072) showed that the expression levels of GPRC5A was high and PTGES was low in normal human lung tissue; whereas the expression levels of these genes in lung tumor tissues were opposite in (Fig. 7b). Statistically, GPRC5A was inversely correlated with PTGES in mRNA levels (Fig. 7c). Moreover, lung cancer patients with either low GPRC5A or high PTGES expression exhibited poor overall survival (Fig. 7d, e). Taken together, these results suggest that repressed GPRC5A and upregulated PTGES predicts poor overall survival in patients with NSCLCs.

## Discussion

*Gprc5a*-ko mice provide an animal model that links inflammatory response and tumorigenesis/metastasis. *Gprc5a*-ko mouse lungs are susceptible to inflammation, spontaneous and carcinogen-induced tumorigenesis [24, 27], which resembles the pathological features in human. In this study, we show that PTGES/PGE_2_ signaling promotes lung metastasis via two distinct mechanisms. First, PTGES/PGE_2_ signaling intrinsically endows tumor cells resistant to T-cell cytotoxicity. Second, PTGES/PGE_2_ signaling induces immunosuppression extrinsically via inducing MDSC recruitment and polarization of M2 macrophages (Fig. 7f).

Previously, PTGES/PGE_2_ was shown to induce PD-L1 expression in macrophages and MDSC [22]. However, we did not found that PD-L1 was significantly suppressed in 1601-ko-Ptges cells compared to control 1601-NS cells. This suggests other mechanisms involved. We find that PTGES/PGE_2_ signaling is essential for the stem-like features, including expression of ABCG2 and EMT-related markers. Consistently, PTGES/PGE_2_ signaling is essential for stem cell-enriched (CD44-positive) subpopulation, migration and invasion, 3D sphere and colony formation in vitro. Similarly, EMT induced by Snail expression in melanoma cells was found to accelerate metastasis [38]. And, there is a strong correlation between EMT and immunosuppressive target gene expression [39]. Thus, the intrinsic role of PTGES/PGE_2_ signaling in immunosuppression on tumor cells is linked to its role in induction of the EMT program.

COX/Ptges/PGE_2_ signaling has been implicated to inhibit host immunity. However, the detailed mechanism remains controversy. PGE_2_ has been shown to inhibit innate immunity, such as to inhibit the expression of type I interferon in macrophages during influenza A virus (IAV) infection [40], and inhibit NK cell viability and chemokine production in melanoma model [41]. PGE_2_ was considered to render melanoma cells evade immunity via type I interferon [42]. PGE_2_ has also been shown to alter the cytokine profile of T cells, such as to induce the cytokines for Th2 cells (IL-10), and to repress the cytokines for Th1 cells (IL-12 and IFNγ) [43]. Recently, it was shown that COX2/mPGES1(Ptges)/PGE_2_ pathway upregulates PD-L1 expression in tumor-associated macrophages (TAM) and MDSC, which contributes to the immunosuppression in bladder cancer [22]. In addition, upregulated PGE_2_ receptor EP4 was found in COX2-expressing triple-negative breast cancer, whereas targeting EP4 was suggested to benefit the therapeutic effect of PD-L1 inhibitors [44].

In this study, we performed a comprehensive analysis on lung tumor cells and immune cells in lung tissue microenvironment. Our study showed that PGE_2_-mediated immunosppression is mainly through two mechanisms. First, the intrinsic role of the Ptges/PGE_2_ pathway, which confers lung tumor cells resistant to T-cell cytotoxicity. Second, the extrinsic role of PGE_2_ signaling, which inhibits host immunity via inducing M2 macrophage polarization and recruitment of MDSC. Moreover, we found that PGE_2_-induced G-CSF is mainly responsible for increased recruitment of MDSC, which is critical for immunosuppression on T cells; whereas PGE_2_ itself does not directly increase recruitment of MDSC. Our study provides a more detailed mechanism on PGE_2_-mediated immunosuppression.

Previously, many studies were to assess the direct role of PGE_2_ on tumor cells. For example, PGE_2_ released from tumor cells were found to enhance cancer stem cells (CSC), while blockage of PGE_2_ signaling, via PGE_2_-neutralizing antibody and COX inhibitor celecoxib, abrogated CSC repopulation of bladder cancer [18]. In another study, PTGES inhibitor Cay10526 was shown to inhibit subcutaneous melanoma tumor growth in nude mice at high doses (50 mg/kg) [37]. Of notion, the tumor models used in these studies were xenografts in immune-deficient nude mice. Thus, the therapeutic effects were due to the direct role of PTGES/PGE_2_ inhibitor on tumor cells. The impact on host immunity is unclear. In this study, we performed the analysis on *Gprc5a*-ko mice, an immune-competent mouse model of C57 with inflammatory lung. Remarkably, treatment with PTGES inhibitor Cay10526 (5 mg/kg) at a dose ten times lower than that used in that report, for only one week, still significantly inhibited lung metastasis. This suggests that the therapeutic effect through restoration of adaptive immunity by PTGES targeting is much greater than direct cytotoxicity on tumor cells. In support, treatment of animal with Cay10526 at low dose, suppressed MDSCs, restored adaptive immunity, and inhibited lung metastasis, whereas treatment of tumor cells with Cay10526 at dose as high as 50 μM did not show cytotoxic effect on target tumor cells in vitro. Another advantage is that PTGES inhibitor is specific in inhibition of PGE_2_ production, whereas COX2 inhibitors suppress all of PGH_2_-derived products, including PGE_2_, PGD_2_, PGF_2a_, PGI_2_, and TXA_2_ [13]. Long term suppression of PGH_2_-derived products may cause serious side effects.

Recently, tumors are also classified into “hot” and “cold”, which reflects the status of infiltration of T lymphocytes, and the ability to response to immunotherapy. Here, we showed that PTGES is inversely correlated with CD8^+^ T cells in NSCLC samples. This suggests that upregulated PTGES/PGE_2_ signaling is crucial for of CD8^+^ T-cell infiltration. Thus, PTGES/PGE_2_ are the candidate markers for “hot” and “cold”, which may provide a direction for therapeutic application. Taken together, we found that PTGES/PGE_2_ signaling provides a functional link between inflammation and immunosuppression in lung metastasis.

## Materials and methods

### Cell lines and cell culture

HEK293T and HCC827 were tested and authenticated by DNA typing at Shanghai Jiao Tong University Analysis Core. Primary mouse lung cancer cells (SJT-1601) were isolated from NNK-induced lung tumor of *Gprc5a*-ko mouse at the age of 14 months [26]. We constructed the stable cell line SJT-1601-ko-Ptges by CRISPR/Cas9 technology. HCC827 stably transfected with nonspecific (sh-NS) or PTGES shRNA. HEK293T and HCC827 were cultured in DMEM. HCC827 were cultured in RPMI-1640.

### Plasmids, reagents and antibodies

Detailed information is provided in the Supplementary Experimental Procedures.

### Mice experiments

Detailed information is provided in the Supplementary Experimental Procedures.

### Quantitative real-time (Q-PCR) and primers

Cells were lysed using trizol. Total RNA was extracted with RNA Extract Kit (TIANGEN) and cDNA were prepared from 1 μg total RNA using Fast Quant Kit. The Q-PCR analysis was performed on ABI 7300 real-time PCR machine. All Ct values were standardized by β-actin’s Ct value. All primers were listed in Supporting Information, Table S1.

### Western blot analysis

Cells were lysed with RIPA lysis buffer [45]. Experiments were performed as described previously [46]. All antibodies were diluted for use according to manufacturers’ instructions. Finally, Protein expression was detected by chemiluminescence.

### Flow cytometry analysis

Flow cytometry was performed as described previously [47, 48]. The cells were incubated CD44-APC (BD Pharmingen), however the control were incubated with IgG. After incubation, the cells were suspended in PBS for Flow Cytometry analysis. Mice were sacrificed, and lung tissues were scissors cut into a meaty shape and digested. Cells were stained with fluorescently labeled antibodies. Then cells were analyzed by FACS (BD Biosciences). Finally, the data was analyzed in the Flowjo 7.6.1software. All antibodies information was listed in Supporting Information, Table S2.

### Migration and wound healing assay

Experiments were performed as described previously [49]. Fluorescent images were obtained; reported data are counts of migrated cells with experiments performed in triplicate. For the wound healing assay, cells overspread the culture plate scratch the cells. Then the picture captured as needed by Nikon camera.

### Immunohistochemical staining and clinical samples

A tissue microarray composed of tumor and adjacent normal tissue was stained to identify PTGES and GPRC5A proteins. The IHC protocol and score method were performed as previously described [27]. All antibodies were diluted for use according to manufacturers’ instructions. Human lung cancer tissue samples were obtained from Shanghai Chest Hospital, Shanghai Jiao Tong University (Shanghai, CHINA). Shanghai Chest Hospital approved the use of the tumor samples and animals in this study.

### T-cell activation in vitro

Detailed information is provided in the Supplementary Experimental Procedures.

### Mice lung tissues metabonomics

Detailed information is provided in the Supplementary Experimental Procedures.

### Statistical analysis

Comparisons among groups were performed by the Student’s *t*-test or Tukey–Kramer comparison test followed by analysis with GraphPad Prism software (GraphPad Software, San Diego, CA, USA). A *p*-value < 0.05 was considered significant (*p < 0.05; **p < 0.01; ***p < 0.001).

## Supplementary information

supplementary information
