# Supplementary material for: PTGES/PGE2 signaling links immunosuppression and lung metastasis in Gprc5a-knockout mouse model
Source: Oncogene. 2020 Feb 14;39(15):3179–94. doi: 10.1038/s41388-020-1207-6 (PMC7142021; doi:10.1038/s41388-020-1207-6)
Supplement: Supplementary file 1 — supplementary information [file 41388_2020_1207_MOESM1_ESM.pdf]

Supplementary Information

PTGES/PGE_2 Signaling Links Immunosuppression and Lung Metastasis in *Gprc5a*-Knockout Mouse Model

Supplementary Data

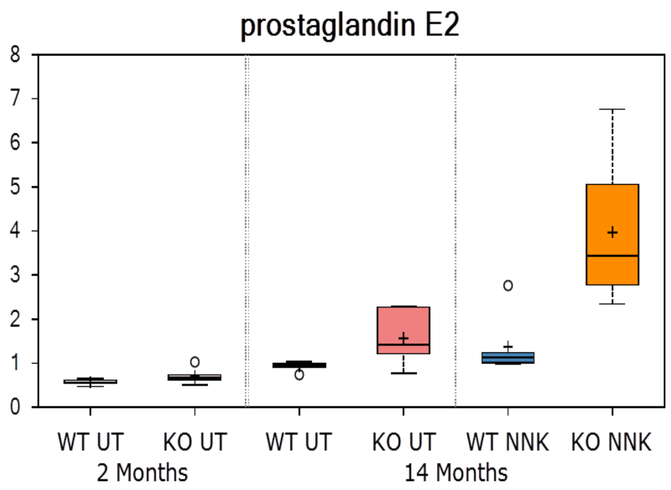

Figure S1. The relative concentration of PGE_2 in mouse lung tissues from the different treatment groups as determined by the metabonomics analysis.

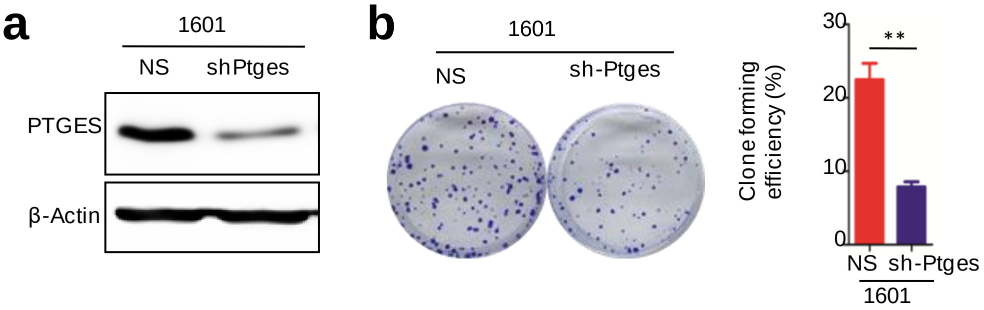

Figure S2. Dysregulation of PTGES is essential for maintaining the increased proliferation of SJT-1601 cells. **a** Western blot analysis of PTGES protein levels in SJT-1601-NS and SJT-1601-shPTGES cells. **b** Colony formation assay of SJT-1601-NS and SJT-1601-shPTGES (mean ± SD from 3 separate experiments). *p <0.05; **p < 0.01; ***p < 0.001.

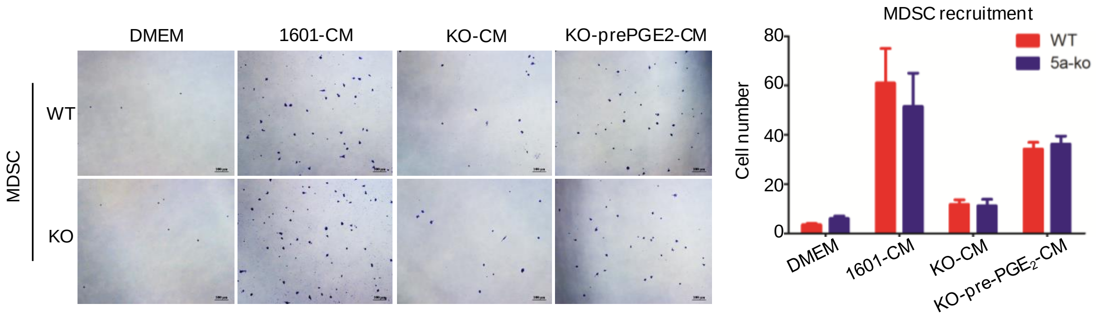

Figure S3. The conditional media (CM) from 1601, 1601-ko-Ptges (KO), or 1601-ko-Ptges treated with PGE_2 (KO-prePGE_2) were used for MDSC recruitment (n=3). *p <0.05; **p < 0.01; ***p < 0.001.

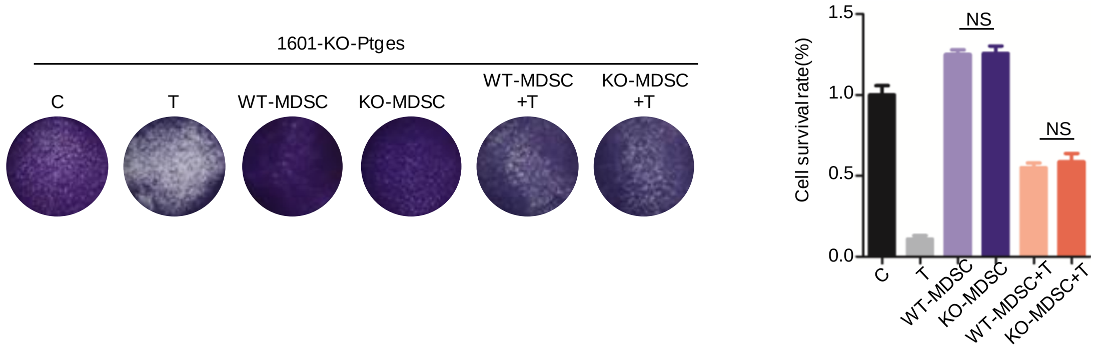

Figure S4. T cell-mediated cytotoxicity was assessed in SJT-1601-ko-Ptges cells with C57-WT and *Gprc5a*-ko MDSCs co-culture.

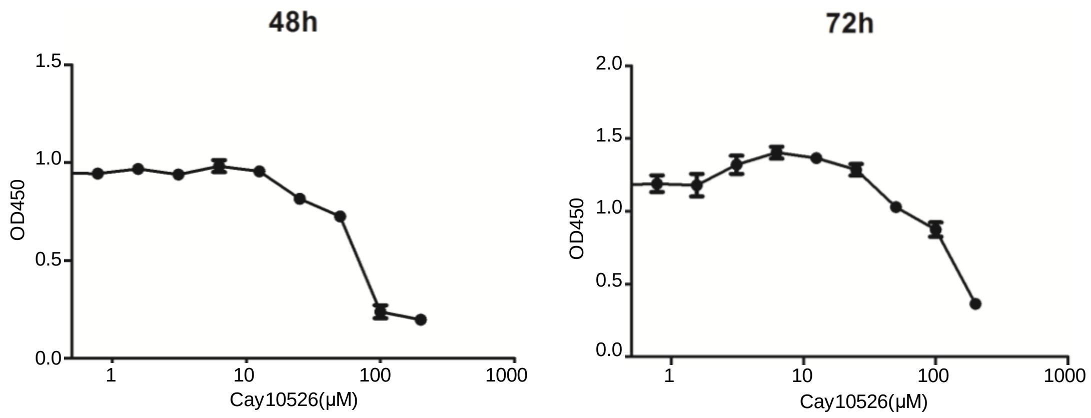

Figure S5. SJT-1601 cells (96-well plate) were treated with Cay10526 as indicated. Cellular viability was assayed using the CCK8 kit.

Supplementary Methods

Plasmids, reagents and antibodies

The following antibodies were used: anti-PTGES (#160140, Cayman chemical), anti-β-actin-HRP (#60008, Proteintech) and anti-Gprc5a (#sc-98885, Santa Cruz). PTGES (#PA5-33000) for IHC were purchased from Thermo fisher. PGE_2 (# p5640) were purchased from Sigma-Aldrich. The Ptges inhibitor Cay10526 (#10010088) was purchased from Cayman chemical. ELISA kit (EMC45) for detecting PGE_2 in mice lung tissues was purchased from ExCell Bio (China). We detected the secretory factors in cell culture supernatants by Bio-plex MAGPIX System. Bio-Plex Pro Mouse Cytokine Grp Panel 23-plex was purchased from Wayen Biotechnologies (Shanghai). Oligonucleotides corresponding to these guide sequences were cloned into the BbsI site of pX330, a bicistronic expression vector encoding both Cas9 and the sgRNA.

Mice experiments

Gprc5a-ko mice were from Dr. R. Lotan (University of Texas M.D. Anderson Cancer Center). Eight-week-old *Gprc5a*-wt and *Gprc5a*-ko mice were received 2 weekly i.p. injections of NNK (100 mg/kg). Twelve months later, mice were sacrificed, some was fixed in paraffin for H&E staining analysis, and the rest lung tissues were homogenated in liquid nitrogen for extraction of protein and RNA. SJT-1601 and ko-Ptges were injected subcutaneously (2×10^5 cells) in nude and C57BL/6 mice. The tumor volume was measured thrice a week. The tumor volume was calculated. Then, xenografts consisting of 5×10^5 tumor cells were injected tail vein in nude, C57BL/6 and *Gprc5a*-ko mice. About 3 weeks, mice were subjected to killing. We measured the tumor and analyzed the data. For in vivo PTGES inhibitor therapy assay, SJT-1601 cells were injected into the tail vein (5×10^5/0.2 mL cells in PBS) of *Gprc5a*-ko mice, one week later, all mice were randomly divided into two groups, 5 mg/kg dose of Cay10526 or control PBS were i.p. injected every day for one weeks, these animals

were sacrificed and lung metastasis were assessed after 3 weeks. All experimental mice were female, at age of 8 weeks. Animal studies were conducted following the guidelines of the Experimental Animal Ethics Committee of Shanghai Jiao Tong University and were approved by the Experimental Animal Ethics Committee of Shanghai Jiao Tong University.

T cell activation in vitro

2 months C57-WT mice were sacrificed, and single cell suspension from spleen was prepared through 70μm and 40μm nylon mesh after sufficient grinding. $CD8^+$ T cells were obtained by $CD8^+$ T Cell Isolation Kit (Miltenyi Biotec) and were activated with CD3/CD28 antibody conjugated beads (#11453D, Thermo Fisher Scientific) for 3 days. To analyze the killing of tumor cells by T cell inactivation, we co-cultured SJT-1601 and SJT-1601-ko-Ptges cells with T cells according to the ratio of tumor cells: T cells = 1:3 for 3 days in 24 well plates, then the wells were washed twice with PBS to remove T cells. The surviving tumor cells were fixed and stained with crystal violet solution.

Mice lung tissues metabonomics

Metabonomics profiling analysis of mouse lung tissues were conducted using the Metabolon platform. Six mouse groups were used: 2 months *Gprc5a*-wt; 2 months *Gprc5a*-ko; 14 months *Gprc5a*-wt; 14 months *Gprc5a*-ko; 14 months *Gprc5a*-wt treated with NNK; 14 months *Gprc5a*-ko treated with NNK. There were 6 mice per group. Lung tissues harvested from the above groups were quenched in liquid nitrogen, and the samples were transferred to Metabolon Inc. (Durham, NC) for metabonomics analysis. To identify metabolites, the platform used ultrahigh performance liquid chromatography/tandem mass spectrometry (UHPLC/MS-MS) and gas chromatography/mass spectrometry (GC/MS) to identify features in the experimental samples against a reference library of chemical standards that include molecular weight, retention time and MS spectra.

Supplementary Table S1. Primers used in the study

Primers for Real-time PCR		sequence (5′→.3′)
Ptges	F	GGATGCGCTGAAACGTGGA
	R	CAGGAATGAGTACACGAAGCC
m-M1-TNFα	F	CAGGCGGTGCCTATGTCTC
	R	CGATCACCCCGAAGTTCAGTAG
m-M1-IL-6	F	TAGTCCTTCCTACCCCAATTTCC
	R	TTGGTCCTTAGCCACTCCTTC
m-M2-Fizzl	F	CCTGCTGGGATGACTGCTA
	R	TGGGTTCTCCACCTCTTCAT
m-M1-IL-12α	F	ACTCTGCGCCAGAAACCTC
	R	CACCCTGTTGATGGTCACGAC
m-M1-CXCL10	F	CCAAGTGCTGCCGTCATTTTC
	R	GGCTCGCAGGGATGATTTCAA
m-M2-Arg1	F	TGGCTTGCGAGACGTAGAC
	R	GCTCAGGTGAATCGGCCTTTT
m-M2-MRC1	F	CTCTGTTCAGCTATTGGACGC
	R	CGGAATTTCTGGGATTCAGCTTC
cas9-Ptges-gRNA1	F	CACCGGGAATGAGTACACGAAGCCGAGG
	R	AAACCCTCGGCTTCGTGTACTCATTCCC
cas9-Ptges-gRNA2	F	CACCGGAACCCACGCCTTCGCTCCGGGG
	R	AAACCCCCGGAGCGAAGGCGTGGGTTCC

β-actin	F	AACAGTCCGCCTAGAAGCAC
	R	CGTTGACATCCGTAAAGACC
h-shNS		CAACAAGATGAAGAGCACCAA
h-shPTGES		TGGATGCACTTCCTGGTCT
m-shPtges		CACACTCCCTCTTAACCAT

Supplementary Table S2. Antibodies information for flow cytometry analysis

Antibodies	Art.No.	Brand
Fixable Viability Dye eFluor™ 780	65-0865-14	ebioscience
PerCP/Cyanine5.5 anti-mouse CD45	103132	Biolegend
Brilliant Violet 605™ anti-mouse Ly-6C	128035	Biolegend
PE-Cy™7 Rat Anti-Mouse CD3	560591	BD
BV510 Rat Anti-Mouse CD4	563106	BD
BV421 Rat Anti-Mouse CD8a	563898	BD
PE Rat Anti-CD11b	553311	BD
PE-CF594 Rat Anti-Mouse Ly-6G	562700	BD
BUV395 Hamster Anti-Mouse CD11c	564080	BD
Alexa Fluor® 647 Rat Anti-Mouse F4/80	565854	BD
APC Mouse Anti-Mouse NK-1.1	561117	BD
